# Hippocampal Deletion of BDNF Gene Attenuates Gamma Oscillations in Area CA1 by Up-Regulating 5-HT3 Receptor

**DOI:** 10.1371/journal.pone.0016480

**Published:** 2011-01-26

**Authors:** Ying Huang, Alexei Morozov

**Affiliations:** 1 Unit on Behavioral Genetics, Laboratory of Molecular Pathophysiology, National Institute of Mental Health, National Institutes of Health, Bethesda, Maryland, United States of America; 2 Department of Physiology and Pathophysiology, Shanghai Medical College, Fudan University, Shanghai, China; Chiba University Center for Forensic Mental Health, Japan

## Abstract

**Background:**

Pyramidal neurons in the hippocampal area CA3 express high levels of BDNF, but how this BDNF contributes to oscillatory properties of hippocampus is unknown.

**Methodology/Principal Findings:**

Here we examined carbachol-induced gamma oscillations in hippocampal slices lacking BDNF gene in the area CA3. The power of oscillations was reduced in the hippocampal area CA1, which coincided with increases in the expression and activity of 5-HT3 receptor. Pharmacological block of this receptor partially restored power of gamma oscillations in slices from KO mice, but had no effect in slices from WT mice.

**Conclusion/Significance:**

These data suggest that BDNF facilitates gamma oscillations in the hippocampus by attenuating signaling through 5-HT3 receptor. Thus, BDNF modulates hippocampal oscillations through serotonergic system.

## Introduction

Brain-derived neurotrophic factor has multiple neuroregulatory functions [1], [2], [3], [4] and one of its major targets are GABAergic neurons [5], [6], which play essential role in oscillatory activity of neuronal networks [7], [8], [9]. Among several types of brain oscillations, gamma oscillations, which include frequencies ranging from 25 to 100 Hz [10], draw a lot of attention, because they are considered an integrating mechanism, which couples different brain structures during memory encoding and retrieval [11], [12], [13]. In addition, changes in gamma oscillatory activity in human brain have been found in several psychiatric illnesses including schizophrenia and bipolar disorder [14], [15].

Hippocampus is one of the brain areas generating gamma oscillations, which are driven by a network of connected inhibitory neurons and require GABAergic transmission [7], [16]. Fast-spiking parvalbumin-positive interneurons in particular are thought to be responsible for driving gamma oscillations [9], [17] and genetic attenuation of excitatory inputs in these neurons have been shown to reduce oscillation power in the hippocampal area CA3 [18].

Hippocampus, where oscillations are thought to be involved in cognition and memory [19]–[20], expresses high levels of BDNF [21], which has been proposed to modulate oscillations by influencing the firing of GABAergic neurons [22]. Nevetherless, experimental evidence that BDNF modulates gamma oscillations via inhibitory neurons is lacking.

In the hippocampus, BDNF is highly expressed in CA3 pyramidal neurons, which appear to deliver BDNF protein to area CA1 via the Schaffer collateral axons [21]. Given that BDNF influences firing of GABAergic neurons, which are required for gamma oscillations in CA3 and CA1 [23], we hypothesized that BDNF may modulate these oscillations. To test this hypothesis, we examined carbachol-induced gamma oscillations in hippocampal slices from conditional BDNF knockout mice lacking BDNF gene in the CA3 pyramidal neurons (KO mice). In these slices, the oscillation power was reduced in CA1, but not CA3, when compared to slices from wild type mice; yet, this reduction was partially reversed in the presence of tropisetron, an inhibitor of 5-HT3 receptor, whose expression was elevated in KO mice.

## Results

### Power of gamma oscillations in area CA1, but not CA3 is reduced in BDNF KO mice

Mice used in this study lack BDNF gene in the hippocampal area CA3, but not in CA1 or dentate gyrus; the expression level of BDNF in these animals has been characterized in a separate study and was reduced in the whole hippocampus of 6–7 week old mice by more than 25% (manuscript submitted to GBB).

**Figure 1:**
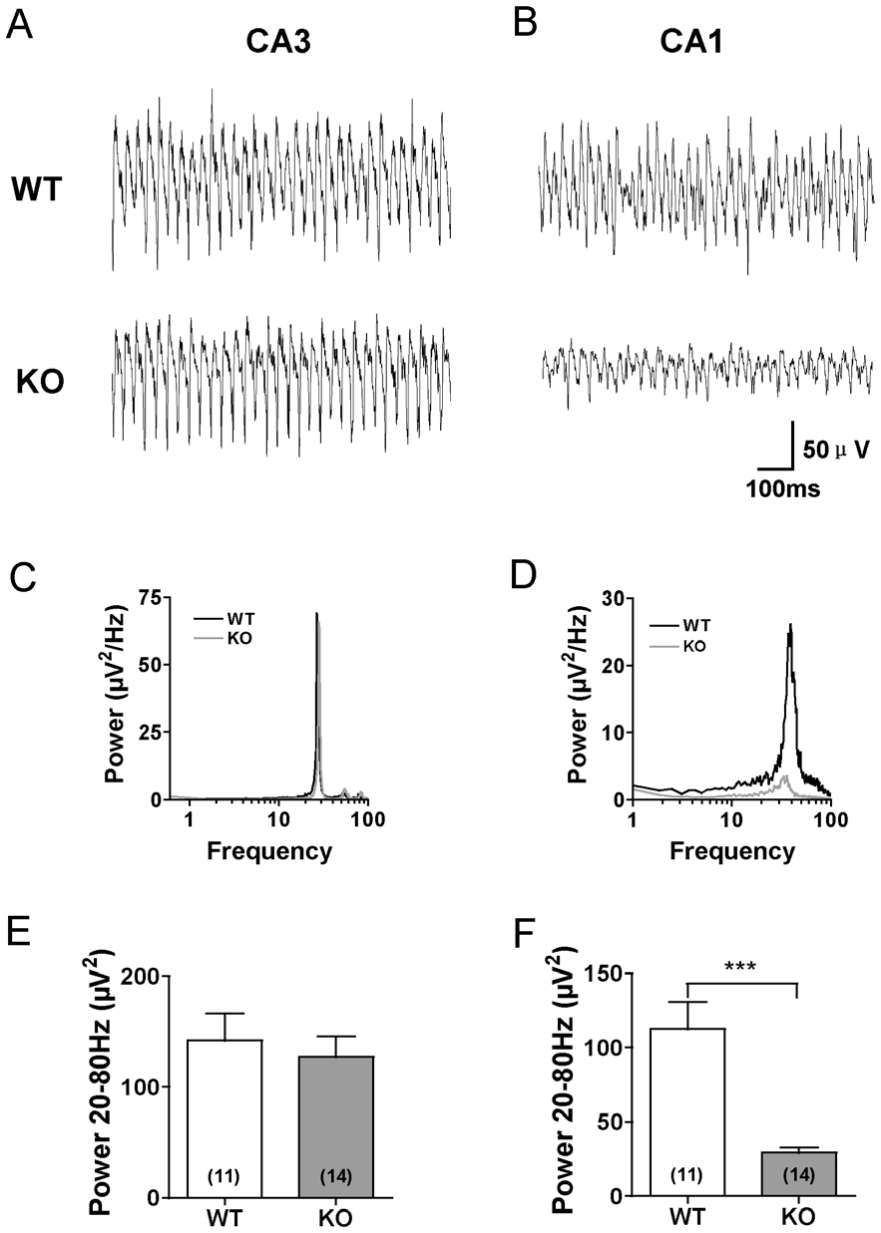
Power of gamma oscillations is reduced in area CA1 but not CA3 in slices from KO mice. A–B. Example traces of field oscillations induced by 25 µM carbachol in hippocampal areas CA3 (A) and CA1 (B). C–D. Power spectra for oscillations in A (C) and B (D). E–F. Pooled data for integral oscillation power (20–80 Hz) in area CA3 (E) and CA1 (F). ***p<0.001. Data represent mean ± SEM.

Bath application of carbachol (25 µM) in hippocampal slices from WT and KO mice induced gamma oscillations in areas CA3 and CA1 with peak frequency of 31.00±1.17 Hz and 34.40±0.98 Hz respectively. In the presence of carbachol, oscillations reached maximum power and steady state in about 4–5 min and lasted during the entire recording period of 30 min. Longer recording sessions confirmed stability of oscillations for at least 60 min (data not shown). The integral 20–80 Hz power of oscillations in area CA3 did not differ significantly between genotypes (WT: 141.84±24.28 µV^2^, n = 13; KO: 126.89±18.7 µV^2^, n = 11, p>0.05) (Fig. 1A, C, E). In contrast, oscillation power in area CA1 was significantly lower in slices from KO than in WT mice (WT: 112.83±17.85 µV^2^, n = 11; KO: 29.34±3.30 µV^2^, n = 14, p<0.001) (Fig. 1B, D, F).

### Elevated 5-HT3 receptor-mediated GABAergic transmission in BDNF KO mice

Oscillations in the brain strongly depend on GABAergic neurons [7], [24], whose activity is modulated by serotonergic system, which, in turn is regulated by BDNF [25]. Given the evidence that serotonergic system modulates oscillations, including gamma rhythm [26], we hypothesized that BDNF augments gamma oscillations in area CA1 by influencing serotonergic and GABAergic transmission.

In search for possible changes in GABAergic transmission in KO mice, we recorded spontaneous inhibitory postsynaptic currents (sIPSC) in CA1 pyramidal neurons in the absence and presence of 5-HT, which enhances sIPSC in these areas by acting on 5-HT2 and 5-HT3 receptors of GABAergic neurons [27] [28], [29].

**Figure 2:**
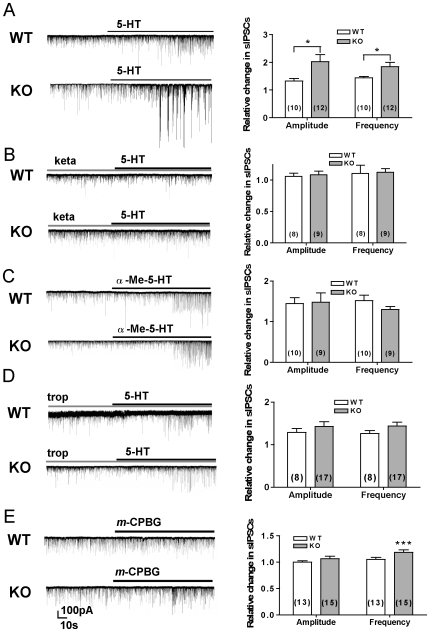
Enhancement of 5-HT-induced sIPSC in pyramidal neurons of CA1 of KO mice is mediated by 5-HT3 receptors. A–E. Pharmacological modulation of sIPSC. Left: Examples of sIPSC traces. Right: summary data. sIPSC modulation by 20 µM 5-HT (A), by 5-HT in the presence of 20 µM 5-HT2 receptors antagonist ketanserin (keta) (B), by 20 µM 5-HT2 receptors agonist α-methyl-5-HT (α-Me-5-HT) (C), by 5-HT in the presence of 30 nM 5-HT3 receptor antagonist tropisetron (trop) and by 1 µM 5-HT3 receptor agonist *m*-chlorophenyl biguanide (*m*-CPBG). Number of cells recorded from is indicated in parenthesis. *p<0.05, **p<0.01. Data represent mean ± SEM.

Bath application of 5-HT (20 µM) increased amplitude and frequency of sIPSC in both genotypes (p<0.05) and these increases were higher in slices from KO than WT mice (fold increase; amplitude, WT: 1.33±0.09, n = 10, KO: 2.02±0.25, n = 12, p<0.05; frequency, WT: 1.44±0.05, KO: 1.84±0.15, p<0.05, when compared between genotypes) (Fig. 2A).

To identify serotonin receptor(s) responsible for the differences between genotypes, we modulated 5-HT2 and 5-HT3 receptors. Pre-treatment of slices with a 5-HT2 receptors antagonist, ketanserin (20 µM), abolished potentiation of sIPSC by 5-HT in both genotypes (amplitude and frequency, WT: n = 8, KO: n = 9, p>0.05, compared to baseline) (Fig. 2B), indicating that 5-HT2 receptor is necessary for 5-HT-induced sIPSC. Application of a 5-HT2 receptor agonist α-methyl-5-HT (α-Me-5-HT) (20 µM) enhanced sIPSC in both genotypes to a similar degree (fold increase; amplitude, WT: 1.45±0.14, n = 10, KO: 1.48±0.23, n = 9, p>0.05; frequency, WT: 1.52±0.14, KO: 1.30±0.07, p>0.05) (Fig. 2C), suggesting no difference between genotypes in the function of 5-HT2 receptors with respect to sIPSC.

A selective 5-HT3 receptors antagonist tropisetron (30 nM) attenuated the 5-HT potentiation more strongly in KO than in WT group and diminished the difference between genotypes in the effect of 5-HT on sIPSC (fold increase; amplitude, WT: 1.29±0.09, n = 8, KO: 1.43±0.11, n = 9, p>0.05; frequency, WT: 1.26±0.07, KO: 1.44±0.09, p>0.05) (Fig. 2D). Meanwhile, a selective 5-HT3 receptor agonist m-chlorophenyl biguanide (m-CPBG) (1 µM) increased sIPSC frequency in KO (1.19±0.05, n = 15, p<0.01, compared to baseline), but not in WT group (1.00±0.03, n = 13, p>0.05) (Fig. 2E), suggesting that the function of 5-HT3 receptor with respect to sIPSCs is enhanced in KO mice.

**Figure 3:**
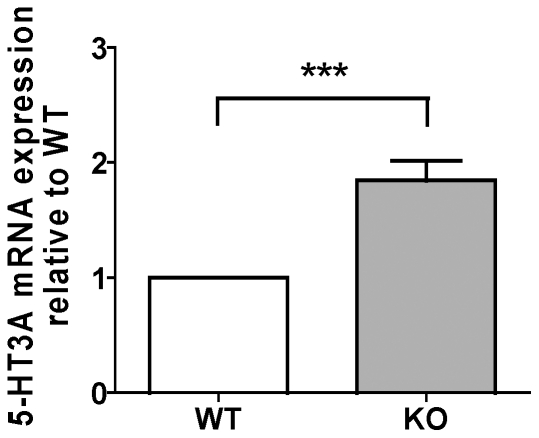
Increase in the expression of 5-HT3a mRNA in the hippocampus of KO mice. mRNA expression of 5-HT3a receptor in KO mice relative to that in WT as determined by RT-PCR. ***p<0.001.

Upregulation of 5-HT3 receptor was further confirmed at the expression level. Real time PCR (RT-PCR) analysis using total hippocampal mRNA revealed higher expression of the 5-HT3A subunit in KO than in WT mice (fold difference relative to WT, KO: 1.85±0.17, n = 12, p<0.001, one sample t-test) (Fig. 3).

### 5-HT3 receptor block reverses gamma oscillations in KO mice

**Figure 4:**
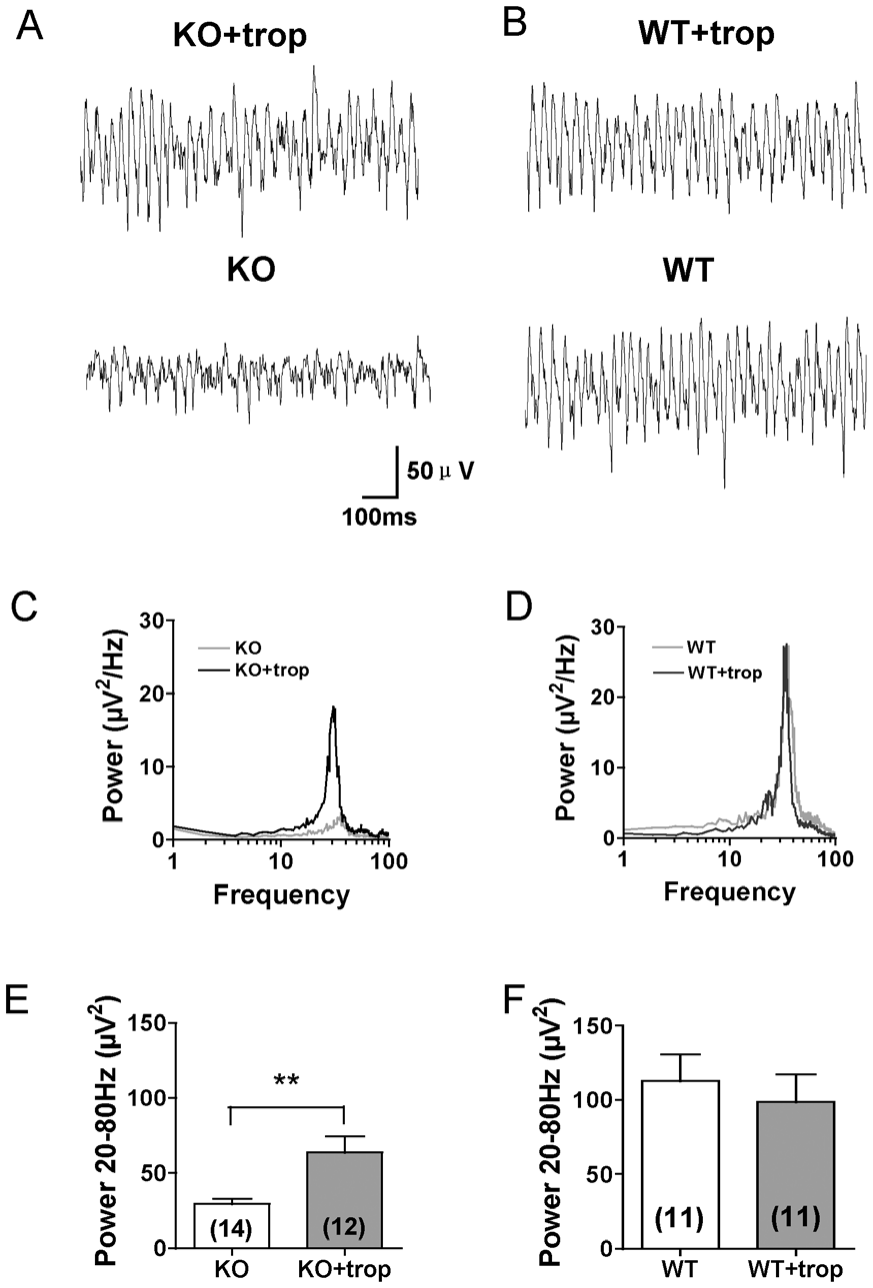
Reduction of gamma oscillation power in KO mice is partially reversed by 5-HT3 receptor antagonist tropisetron. A–B. Upper: Example traces of field oscillations in hippocampal area CA1 induced by carbachol in the presence of 30 nM tropisetron in slices from KO (KO+trop) (A) and WT (WT+trop) mice (B). Lower: Traces of oscillations obtained in the absence of tropisetron, are shown for comparison. C–D. Power spectra for oscillations in A (C) and B (D). E–F. Pooled data for integral oscillation power (20–80 Hz) in area CA1 in the presence of tropisetron (grey bars); data from Fig. 1 obtained in the absence of tropisetron are shown for comparison (white bars). Number of cells recorded from is indicated in parenthesis. **p<0.01. Data represent mean ± SEM.

To determine whether 5-HT3 receptor inhibits gamma oscillations in area CA1 in KO mice, we examined the effect of 5-HT3 receptors antagonist tropisetron (30 nM) on gamma oscillations. In slices from KO mice, tropisetron partially reversed the oscillation deficiency. The integral oscillation power (20–80 Hz) in tropisetron treated slices was 63.97±10.52 µV^2^ (n = 12), which was significantly higher than in control slices (29.34±3.30 µV^2^, n = 14, p<0.01) (Fig. 4A, C, E). Meanwhile, in slices from WT mice, tropisetron did not change the power of oscillations (p>0.05) (Fig. 4B, D, F). These results indicate that, while normal activity of 5-HT3 receptor does not interfere with gamma oscillations in our system, the upregulation of the receptor in KO mice is partially responsible for the reduction in oscillation power.

**Figure 5:**
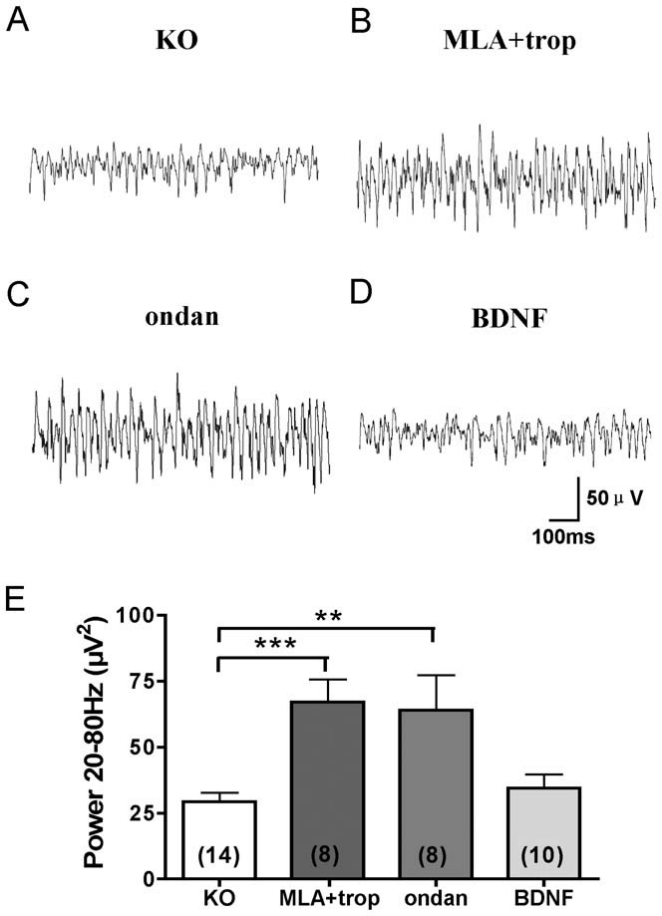
Rescue of gamma oscillations in KO mice is achieved by blocking 5-HT3 receptor, but not by recombinant BDNF. A–D. Example traces of field oscillations in hippocampal area CA1 induced in slices from KO mice by carbachol without other drugs (A), in the presence of 30 nM tropisetron and 50 nM MLA (B), with 30 nM ondansetron (ondan) (C), and with 40 ng/ml BDNF (D). E. Pooled data for integral oscillation power (20–80 Hz) under conditions in A–D as indicated. Number of cells recorded from is shown in parenthesis. **p<0.01, ***p<0.01. Data represent mean ± SEM.

Besides being a specific antagonist of 5-HT3 receptor, tropisetron can also activate the alpha-7 nicotinic receptor [30], [31]. To rule out a possibility that the reversal of oscillation deficit in KO mice was achieved by activation of the alpha-7 nicotinic receptor (α-7nAChR), we re-examined the effect of tropisetron in the presence of α-7 nAChR antagonist methyllycaconitine (MLA, 50 nM). MLA did not prevent tropisetron from rescuing gamma oscillations in slices from KO mice (the integral oscillation power (20–80 Hz): 66.97±8.60 µV^2^, n = 8, p<0.001, compared with the slices from KO mice (Fig. 5B, E). Moreover, a 5-HT3 receptor antagonist ondansetron (30 nM), which is not known to activate α-7 nAChR, showed the same rescuing effect on oscillations as tropisetron (integral oscillation power (20–80 Hz): 63.91±13.37 µV^2^, n = 8, p<0.01, compared with the slices from KO mice (Fig. 5C, E). These data indicate that gamma oscillations in slices from KO mice were rescued by inhibition of 5-HT3 receptor.

To examine whether oscillation deficit in KO slices could be reversed by acute application of recombinant BDNF protein, we co-applied carbachol in the presence of BDNF. BDNF (40 ng/ml) did not rescue the oscillation deficit (the integral oscillation power (20–80 Hz): 34.39±5.20 µV^2^, n = 10, p>0.05, compared with the slices form KO mice (Fig. 5D, E), suggesting that changes caused by BDNF KO could not be reversed acutely in the slice.

## Discussion

Hippocampal gamma oscillations originate in area CA3 and propagate to area CA1 [12], [24]. Our study suggests that BDNF from CA3 pyramidal neurons does not affect these oscillations in CA3, but facilitates them in CA1 by attenuating expression and activity of 5-HT3 receptor, which is expressed in a subpopulation of PV-negative GABAergic neurons, but not in the principal neurons [32], [33].

Several observations suggest that BDNF facilitates oscillatory activity. In cultured hippocampal neurons, it increases fidelity of spikes during periodic current injections [34] and facilitates spontaneous Ca2+ oscillations [35]; in developing cortical neurons, it potentiates frequency of synchronous spontaneous oscillations [36]. BDNF also modulates GABAergic transmission [5] [37], which underlies oscillatory activities, but, to our knowledge, there has been no direct evidence so far that BDNF influences oscillations through GABAergic neurons. The present study provides such evidence and suggests that the effect is mediated by serotonergic system.

First, gamma oscillations were attenuated in slices from KO mice. Second, in these slices, 5-HT enhanced sIPSCs more strongly than in control slices, but this difference was attenuated by a HT3 receptor antagonist, which also reversed the oscillation deficit. These data suggest that the up-regulation of 5-HT3 receptor and subsequent changes in GABAergic neurons, which express this receptor, were responsible for attenuated oscillations in slices from KO mice.

However, the reversal of oscillation deficit by 5-HT3 receptor antagonist was only partial, possibly because of irreversible changes caused by chronic up-regulation of 5-HT3 receptor. The partial rescue may also indicate that BDNF regulates gamma oscillations through additional 5-HT3 receptor-independent mechanisms, for example, by directly modulating fast-spiking interneurons [22], [38], [39]. We could not reverse the oscillation deficit by acute application of recombinant BDNF. It is consistent with the idea that effect of BDNF on gamma oscillations is indirect and may be mediated by molecules like 5-HT3 receptor, whose expression or activity are altered during long-lasting reduction in BDNF concentration in KO mice and cannot be reversed rapidly.

Surprisingly, in contrast to the oscillation deficit in CA1, slices from KO animals had normal oscillations in CA3, despite the deletion of BDNF gene in this area. This difference may result from the lower serotonergic innervations of the CA3 pyramidal layer [40], which may render CA3 oscillations less dependent on 5-HT than the oscillations in area CA1, which has more serotonergic axons. Such explanation is consistent with an idea that the main effect of BDNF on oscillations is mediated by serotonergic system.

Changes in gamma oscillations have been found in several brain illnesses, including schizophrenia, bipolar disorder and Alzheimer [14], [15], [41], [42], some of which include cognitive impairments. On the other hands, BDNF has been implicated in cognitive function, because it regulates synaptic plasticity [43] and animals with deletions of BDNF gene in brain areas that include hippocampus show deficits in synaptic plasticity [44] and cognitive impairments [45], [46], whereas animals with increased expression of BDNF show better performance in cognitive tasks [47]. Modulation of oscillatory activity by BDNF could be one of the mechanisms responsible for those behavioral changes.

## Materials and Methods

### Generation of mice with BDNF gene deletion in CA3 pyramidal neurons

All experiments were performed under the Animal Study Protocol LMP 10-09 approved by the National Institute of Mental Health Animal Care and Use Committee. Male mice with homozygous floxed BDNF gene [44] carrying G32-4 allele of Cre-recombinase [48] (BDNF *^ff, Cre^* animals) were crossed with BDNF *^ff^* females, which yielded BDNF *^ff, Cre^* mice further referred to as KO, and BDNF *^ff^* mice referred to as WT. The presence of Cre and floxed BDNF alleles was determined as previously described [44]. Only male mice were used and all animals were on C57BL/6J background.

### Electrophysiology

Mice (6–7 week old) were decapitated under isoflurane anesthesia. Brains were rapidly removed and immersed in ice-cold cutting solution containing, in mM: 252 sucrose, 2.5 KCl, 4 MgCl_2_, 0.5 CaCl_2,_ 1.2 NaH2PO4, 26 NaHCO3, 10 Glucose, saturated with 95% O2 and 5% CO2. 400 µm horizontal slices were prepared using Microslicer (DSK, Kyoto, Japan). The slices were incubated in ACSF containing, in mM: 124 NaCl, 3.5 KCl, 2 MgCl_2_, 2 CaCl_2,_ 1.25 NaH2PO4, 25 NaHCO3, 10 Glucose, saturated with 95% O2 and 5% CO2 at room temperature for at least 1 h before recording.

Slices were transferred to a submerged two surface superfused recording chamber at 33°C [49] and perfused at a rate of 4.5–5.5 ml/min with ACSF identical to the incubation solution except for the concentration of MgCl_2_ which was 1.5 mM. Recordings were performed using a Muticlamp 700B amplifier (Molecular Devices, Sunnyvale, CA). Field oscillations were recorded using 2.5–3.5 MΩ glass pipettes filled with ACSF. For the whole cell recordings, the pipette resistance was 3–5 MΩ and the internal solution contained (in mM): 140 KCl, 10 HEPES, 1 EGTA, 2 MgCl_2_·6H_2_O, 2 Na_2_ATP, 0.4 Tris GTP (pH adjusted to 7.3 by KOH), 5 QX-314, and ACSF included 6-cyano-7-nitroquinoxaline-2,3-dione (CNQX) (20 µM) and D-APV (10 µM). Cells were clamped at −70 mV, series resistance was ≤25 MΩ, and the data were discarded when Rs value changed by more than 20%. GABA_A_ nature of the inward synaptic currents was verified by their block with picrotoxin (200 µM). Current responses were analyzed with Mini Analysis v6.0 software (Synaptosoft, Fort Lee, NJ). The power spectra and integral power for 20–80 Hz frequency range were calculated for 60 second long recording segments using fast Fourier transformation in Clampfit 9.2 (Molecular Devices).

### Quantitative real time PCR

Total RNA from whole hippocampi was isolated using RNeasy Lipid Tissue Mini Kit (Qiagen, Valencia, CA) and reverse transcribed (2 µg) using SuperScript III First Strand-Synthesis System For RT-PCR (Invtirogen, Carlsbad, CA). PCR was performed using Smart Cycler and data were analyzed using Smart Cycler System Software (Cepheid, Sunnyvale, CA). cDNA was amplified using FastStart Taq DNA Polymerase (Roche, Mannheim, Germany) in the presence of SYBR Green I (Invtirogen) at 1: 40,000 dilution. Primers for GAPDH (forward: 5′-AATGTGTCCGTCGTGGATCTGA-3′; reverse: 5′-GATGCCTGCTTCACCACCTTCT-3′) and 5-HTr3A (forward: 5′-TGGACTCCTGAGGACTTCGACAAT-3′; reverse: 5′-′TGAACTTCACCTCGATGATGCACG-3′) were at 500 nM. The cycling conditions were: 5 min at 95°C followed by 40 cycles of 95°C/15 sec, 60°C/30 sec. The cycle threshold (Ct) was determined as the zero for the second derivative of the growth curve function. The fold change in mRNA expression of receptors was determined using the comparative Ct method [50] with GAPDH mRNA as the normalization control.

### Statistical methods

Data are presented as mean ± SEM. Comparisons were performed using two-tail unpaired t-test (if not specified), one sample t-test (real time PCR).

## References

[1] Binder DK, Scharfman HE (2004). Brain-derived neurotrophic factor.. Growth Factors.

[2] Jin X, Hu H, Mathers PH, Agmon A (2003). Brain-derived neurotrophic factor mediates activity-dependent dendritic growth in nonpyramidal neocortical interneurons in developing organotypic cultures.. J Neurosci.

[3] Poo MM (2001). Neurotrophins as synaptic modulators.. Nat Rev Neurosci.

[4] Tanaka J, Horiike Y, Matsuzaki M, Miyazaki T, Ellis-Davies GC (2008). Protein synthesis and neurotrophin-dependent structural plasticity of single dendritic spines.. Science.

[5] Abidin I, Eysel UT, Lessmann V, Mittmann T (2008). Impaired GABAergic inhibition in the visual cortex of brain-derived neurotrophic factor heterozygous knockout mice.. J Physiol.

[6] Yamada MK, Nakanishi K, Ohba S, Nakamura T, Ikegaya Y (2002). Brain-derived neurotrophic factor promotes the maturation of GABAergic mechanisms in cultured hippocampal neurons.. J Neurosci.

[7] Whittington MA, Traub RD, Jefferys JG (1995). Synchronized oscillations in interneuron networks driven by metabotropic glutamate receptor activation.. Nature.

[8] Traub RD, Whittington MA, Stanford IM, Jefferys JG (1996). A mechanism for generation of long-range synchronous fast oscillations in the cortex.. Nature.

[9] Freund TF, Katona I (2007). Perisomatic inhibition.. Neuron.

[10] Hughes JR (2008). Gamma, fast, and ultrafast waves of the brain: their relationships with epilepsy and behavior.. Epilepsy Behav.

[11] Bragin A, Jando G, Nadasdy Z, Hetke J, Wise K (1995). Gamma (40–100 Hz) oscillation in the hippocampus of the behaving rat.. J Neurosci.

[12] Csicsvari J, Jamieson B, Wise KD, Buzsaki G (2003). Mechanisms of gamma oscillations in the hippocampus of the behaving rat.. Neuron.

[13] Popescu AT, Popa D, Pare D (2009). Coherent gamma oscillations couple the amygdala and striatum during learning.. Nat Neurosci.

[14] Farzan F, Barr MS, Levinson AJ, Chen R, Wong W Evidence for gamma inhibition deficits in the dorsolateral prefrontal cortex of patients with schizophrenia.. Brain.

[15] Ongur D, Lundy M, Greenhouse I, Shinn AK, Menon V (2010). Default mode network abnormalities in bipolar disorder and schizophrenia.. Psychiatry Res.

[16] Whittington MA, Faulkner HJ, Doheny HC, Traub RD (2000). Neuronal fast oscillations as a target site for psychoactive drugs.. Pharmacol Ther.

[17] Traub RD, Jefferys JG, Whittington MA (1997). Simulation of gamma rhythms in networks of interneurons and pyramidal cells.. J Comput Neurosci.

[18] Fuchs EC, Zivkovic AR, Cunningham MO, Middleton S, Lebeau FE (2007). Recruitment of parvalbumin-positive interneurons determines hippocampal function and associated behavior.. Neuron.

[19] van Vugt MK, Schulze-Bonhage A, Litt B, Brandt A, Kahana MJ (2010). Hippocampal gamma oscillations increase with memory load.. J Neurosci.

[20] Kahana MJ (2006). The cognitive correlates of human brain oscillations.. J Neurosci.

[21] Conner JM, Lauterborn JC, Yan Q, Gall CM, Varon S (1997). Distribution of brain-derived neurotrophic factor (BDNF) protein and mRNA in the normal adult rat CNS: evidence for anterograde axonal transport.. J Neurosci.

[22] Holm MM, Nieto-Gonzalez JL, Vardya I, Vaegter CB, Nykjaer A (2009). Mature BDNF, but not proBDNF, reduces excitability of fast-spiking interneurons in mouse dentate gyrus.. J Neurosci.

[23] Bartos M, Vida I, Jonas P (2007). Synaptic mechanisms of synchronized gamma oscillations in inhibitory interneuron networks.. Nat Rev Neurosci.

[24] Fisahn A, Pike FG, Buhl EH, Paulsen O (1998). Cholinergic induction of network oscillations at 40 Hz in the hippocampus in vitro.. Nature.

[25] Martinowich K, Lu B (2008). Interaction between BDNF and serotonin: role in mood disorders.. Neuropsychopharmacology.

[26] Puig MV, Watakabe A, Ushimaru M, Yamamori T, Kawaguchi Y (2010). Serotonin modulates fast-spiking interneuron and synchronous activity in the rat prefrontal cortex through 5-HT1A and 5-HT2A receptors.. J Neurosci.

[27] Ropert N, Guy N (1991). Serotonin facilitates GABAergic transmission in the CA1 region of rat hippocampus in vitro.. J Physiol.

[28] Turner TJ, Mokler DJ, Luebke JI (2004). Calcium influx through presynaptic 5-HT3 receptors facilitates GABA release in the hippocampus: in vitro slice and synaptosome studies.. Neuroscience.

[29] Choi IS, Cho JH, Kim JT, Park EJ, Lee MG (2007). Serotoninergic modulation of GABAergic synaptic transmission in developing rat CA3 pyramidal neurons.. J Neurochem.

[30] Macor JE, Gurley D, Lanthorn T, Loch J, Mack RA (2001). The 5-HT3 antagonist tropisetron (ICS 205-930) is a potent and selective alpha7 nicotinic receptor partial agonist.. Bioorg Med Chem Lett.

[31] Papke RL, Porter Papke JK, Rose GM (2004). Activity of alpha7-selective agonists at nicotinic and serotonin 5HT3 receptors expressed in Xenopus oocytes.. Bioorg Med Chem Lett.

[32] Ferezou I, Cauli B, Hill EL, Rossier J, Hamel E (2002). 5-HT3 receptors mediate serotonergic fast synaptic excitation of neocortical vasoactive intestinal peptide/cholecystokinin interneurons.. J Neurosci.

[33] Tecott LH, Maricq AV, Julius D (1993). Nervous system distribution of the serotonin 5-HT3 receptor mRNA.. Proc Natl Acad Sci U S A.

[34] Fujisawa S, Yamada MK, Nishiyama N, Matsuki N, Ikegaya Y (2004). BDNF boosts spike fidelity in chaotic neural oscillations.. Biophys J.

[35] Sakai N, Yamada M, Numakawa T, Ogura A, Hatanaka H (1997). BDNF potentiates spontaneous Ca2+ oscillations in cultured hippocampal neurons.. Brain Res.

[36] Numakawa T, Yamagishi S, Adachi N, Matsumoto T, Yokomaku D (2002). Brain-derived neurotrophic factor-induced potentiation of Ca(2+) oscillations in developing cortical neurons.. J Biol Chem.

[37] Sakata K, Woo NH, Martinowich K, Greene JS, Schloesser RJ (2009). Critical role of promoter IV-driven BDNF transcription in GABAergic transmission and synaptic plasticity in the prefrontal cortex.. Proc Natl Acad Sci U S A.

[38] Itami C, Kimura F, Nakamura S (2007). Brain-derived neurotrophic factor regulates the maturation of layer 4 fast-spiking cells after the second postnatal week in the developing barrel cortex.. J Neurosci.

[39] Berghuis P, Dobszay MB, Sousa KM, Schulte G, Mager PP (2004). Brain-derived neurotrophic factor controls functional differentiation and microcircuit formation of selectively isolated fast-spiking GABAergic interneurons.. Eur J Neurosci.

[40] Mamounas LA, Mullen CA, O'Hearn E, Molliver ME (1991). Dual serotoninergic projections to forebrain in the rat: morphologically distinct 5-HT axon terminals exhibit differential vulnerability to neurotoxic amphetamine derivatives.. J Comp Neurol.

[41] van Deursen JA, Vuurman EF, Verhey FR, van Kranen-Mastenbroek VH, Riedel WJ (2008). Increased EEG gamma band activity in Alzheimer's disease and mild cognitive impairment.. J Neural Transm.

[42] Herrmann CS, Demiralp T (2005). Human EEG gamma oscillations in neuropsychiatric disorders.. Clin Neurophysiol.

[43] Martinowich K, Manji H, Lu B (2007). New insights into BDNF function in depression and anxiety.. Nat Neurosci.

[44] Zakharenko SS, Patterson SL, Dragatsis I, Zeitlin SO, Siegelbaum SA (2003). Presynaptic BDNF required for a presynaptic but not postsynaptic component of LTP at hippocampal CA1-CA3 synapses.. Neuron.

[45] Gorski JA, Balogh SA, Wehner JM, Jones KR (2003). Learning deficits in forebrain-restricted brain-derived neurotrophic factor mutant mice.. Neuroscience.

[46] Heldt SA, Stanek L, Chhatwal JP, Ressler KJ (2007). Hippocampus-specific deletion of BDNF in adult mice impairs spatial memory and extinction of aversive memories.. Mol Psychiatry.

[47] Nakajo Y, Miyamoto S, Nakano Y, Xue JH, Hori T (2008). Genetic increase in brain-derived neurotrophic factor levels enhances learning and memory.. Brain Res.

[48] Nakazawa K, Quirk MC, Chitwood RA, Watanabe M, Yeckel MF (2002). Requirement for hippocampal CA3 NMDA receptors in associative memory recall.. Science.

[49] Hajos N, Ellender TJ, Zemankovics R, Mann EO, Exley R (2009). Maintaining network activity in submerged hippocampal slices: importance of oxygen supply.. Eur J Neurosci.

[50] Bookout AL, Cummins CL, Kramer MF, Pesola JM, Mangelsdorf DJ, Ausubel FM, Brent R, Kingston RE, Moore DD, Seidman JG, Struhl K (2006). High-Throughput Real-Time Quantitative Reverse Transcription PCR.. Current Protocols in Molecular Biology.

